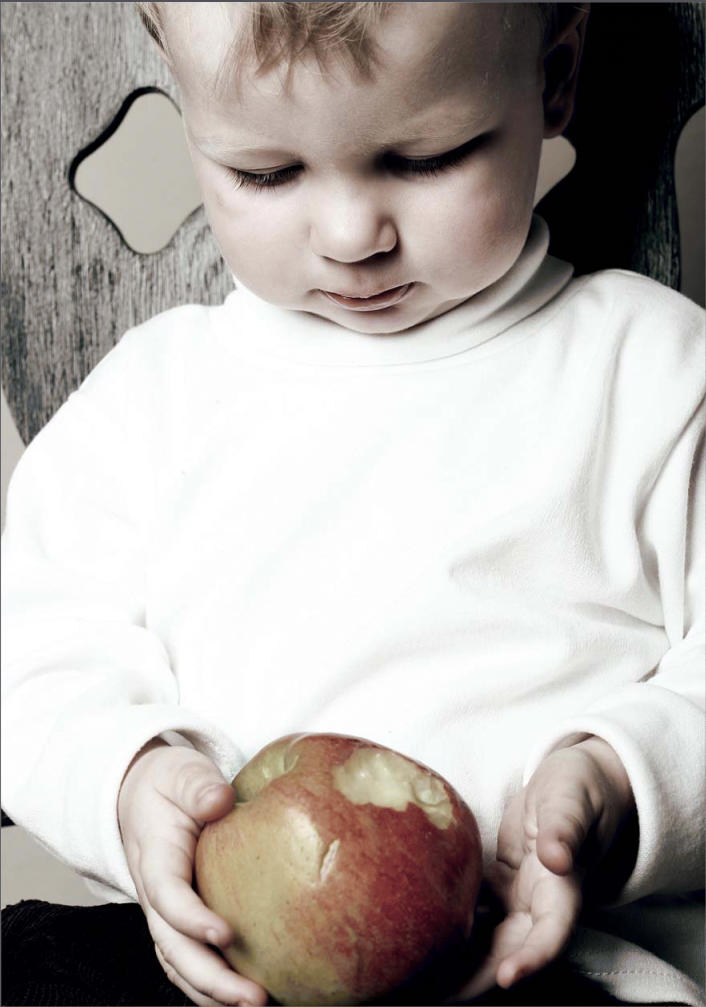# Registering Skepticism: Does the EPA’s Pesticide Review Protect Children?

**DOI:** 10.1289/ehp.114-a592

**Published:** 2006-10

**Authors:** Melissa Lee Phillips

When the EPA announced on 3 August 2006 that it had completed a 10-year review of U.S. pesticide safety, the agency issued a statement full of optimism from administrator Stephen L. Johnson: “By maintaining the highest ethical and scientific standards in its pesticide review, EPA and the Bush administration have planted the seeds to yield healthier lives for generations of American families.”

But Johnson’s words were met with skepticism, not only by environmental activists, but also by some of the EPA’s own scientists. In May, as the agency’s deadline for completing its review neared, nine presidents of unions representing EPA scientists and risk managers had written a letter to the administrator, expressing their concerns that the EPA was about to give approval for organophosphate (OP) and carbamate pesticides that may be neurotoxic, especially in developing fetuses, infants, and children.

“We think there’s a lot of work that remains to be done in terms of getting [adequate] developmental neurotoxicity data,” says William Hirzy, a senior scientist in the EPA’s Office of Toxic Substances and vice president of the National Treasury Employees Union Chapter 280. The union leaders are concerned that the EPA administration is too focused on “avoiding lawsuits from the regulated community,” Hirzy says. Further, in the absence of adequate data, the leaders fear the EPA is making decisions that err on the side of less restriction rather than more precaution.

EPA administrators, however, have responded that they are confident that their assessments are scientifically valid and that no health risks are posed by the pesticides that have been approved for continued use. “We think we have really set a very high bar for pesticide safety in this country,” says Anne Lindsay, deputy director of the EPA Office of Pesticide Programs. “If you are eating food purchased in the U.S., it’s really safe.”

## Two Cancellations

The EPA’s pesticide review began in response to the passage of the 1996 Food Quality Protection Act (FQPA). This act required that the EPA reassess the safety of tolerance levels for food-use pesticide residues in or on raw and processed foods.

The EPA reviewed tens of thousands of new studies in order to decide which pesticides should be banned and which should have new tolerance assessments. These studies came from labs at the EPA, other governmental agencies, and pesticide companies. Over the past decade, the EPA has also developed new risk assessment tools and methods that they are using to better identify chemicals that may be hazardous to human health or the environment. Throughout the research and analysis phase of the review, the EPA also considered opinions from their own advisory committees, as well as from public health watchdog groups and from interested industries. Once all available research had been analyzed, the agency made decisions about each pesticide’s allowed tolerance. After each decision was announced, a 60-day public comment period preceded finalization of the decision.

“The Food Quality Protection Act asked us to take a special look at infants, children, and other subpopulations that might have special sensitivities or susceptibilities,” Lindsay says. The act also asked EPA scientists to examine both aggregate pesticide exposures from food, water, and household uses, as well as exposures to different food-use pesticides that might have cumulative effects in the body.

From 1996 on, all newly registered pesticides had to meet these safety standards, Lindsay says, but there was still the problem of pesticides that had been registered before the FQPA was enacted. So the EPA embarked on a 10-year mission to reassess all food-use pesticides that had not been proven to meet the new requirements. “The idea was to get all tolerances in the U.S. up to this new high safety standard,” Lindsay says.

Congress mandated that the EPA complete all food-use pesticide tolerance reassessment decisions by 3 August 2006. On that date, the EPA announced that it had completed more than 99% of these decisions. The safety reviews still to be completed are those for carbamate pesticides as a class and the carbamate aldicarb in particular. The EPA is currently finishing the assessment of aldicarb, a potent cholinesterase inhibitor. The agency will then be able to issue a review of carbamates as a class, Lindsay says. They’ve already made individual decisions on four other carbamates, proposing to ban carbofuran and limit the use of three others. In all, the EPA evaluated about 230 pesticide active ingredients and 870 inert pesticide ingredients with nearly 10,000 tolerances, according to Lindsay.

In the most recent actions, announced August 3, the EPA opened for public review its proposal to ban not only carbofuran but also lindane, an organochlorine. Carbofuran is an insecticide that is severely toxic to birds. Most carbofuran uses are being canceled immediately, and the remaining uses will be phased out over the next four years. Lindane is used as a seed treatment for several crops. It is known to build up in the environment and in the human body, and is a suspected carcinogen. Most organochlorines, including DDT, were banned in the 1960s and 1970s, and lindane has already been banned in 52 other countries. But in all states except California, lindane is still permitted for use directly on children for the treatment of scabies and lice—an application that is regulated not by the EPA but by the FDA.

## OP Pesticides and Neurotoxicity

Although the EPA’s August decisions proposed complete cancellation only of carbofuran and lindane, while approving many other controversial pesticides, Lindsay points out that the agency had already cancelled numerous pesticides and uses over the 10-year period, most notably on the 32 OP pesticides. A number of these pesticides have been associated with possible cancer effects, fertility problems, or developmental neurotoxicity in animal studies. The primary mechanism through which OP (and carbamate) pesticides work is cholinesterase inhibition: they prevent the breakdown of the neurotransmitter acetylcholine, causing a variety of neurotoxic effects. Although 17 OP pesticides have been cancelled over the past 10 years, many environmental groups—and some EPA scientists—were hoping that the agency would refuse to re-register the others in this class.

“The OP decision, I think, is a bad one,” says Margaret Reeves, a senior scientist with Pesticide Action Network North America. She says her organization recognizes and supports the EPA scientists who sent the letter to Johnson, advising against approval of some of the remaining OP and carbamate pesticides.

According to that letter, too few studies have been done on the developmental neurotoxicity of the remaining food-use OP and carbamate pesticides to make a solid scientific decision about their possible health effects. “[I]n the absence of a robust body of data, FQPA requires EPA to use an additional 10-fold safety factor in its risk assessments when setting pesticide tolerances,” the letter stated. The authors requested that the EPA retain this 10-fold safety assurance “as a precaution when reassessing the tolerances for the remaining OP and carbamate pesticides given the existing uncertainty about developmental neurotoxicity.”

According to Reeves, even that 10-fold measure of safety might not be enough. Several studies have shown there is a large range of vulnerability to OP exposure among different people, especially infants, due to genetic variability in paraoxonase, an enzyme that breaks these chemicals down in the body. “The intraspecies variability is much greater than often considered and much greater than would be [covered by] the FQPA 10-fold factor,” Reeves says.

In a response to the EPA union leaders’ letter, however, EPA acting assistant administrator Susan B. Hazen responded that the absence of an official developmental neurotoxicity study on any given pesticide does not automatically warrant retention of the 10-fold safety factor. “Rather,” she wrote, “EPA should make a judgment, based on the weight of all of the available scientific evidence, to determine what safety factors provide the statutorily required protection for infants and children.”

According to Lindsay, the decision about whether to require a developmental neurotoxicity study of a particular pesticide rests on previous evidence and toxicity data. “We will look at the whole body of evidence that we have,” Lindsay says. “If there are signs the chemical has the capacity to cause neurotoxicity, we would go ahead and ask for a developmental neurotoxicity study to be done.”

Signs that a chemical could be neurotoxic to humans include animal studies that show neurotoxicity, human epidemiological studies that support a causative link between a pesticide and neurological problems, or evidence that the pesticide works through a mechanism already known to be neurotoxic. Says Lindsay, “We think we’re pretty much asking for them when it’s likely they’re going to be needed.”

According to Ray McAllister, regulatory policy and science leader for the trade organization CropLife America, the EPA has done a thorough job of investigating the toxicity of OP and carbamate pesticides. “I don’t think any two groups of pesticides have been more thoroughly investigated by EPA than these two have,” McAllister says. Since 1999, he points out, industry has conducted dozens of developmental neurotoxicity studies on OP and other pesticides, which the EPA took into account in its decision-making process. He adds, “If anything, the approach EPA has taken has been more conservative, more protective, than perhaps they actually need to be, so I don’t think we need to worry about the decision not being protective enough.”

Others aren’t so sure. The authors of the May letter stated they were “concerned that the Agency has not, consistent with its principles of scientific integrity and sound science, adequately summarized or drawn conclusions about the developmental neurotoxicity data received from pesticide registrants.” They cited a January 2006 Inspector General report, *Opportunities to Improve Data Quality and Children’s Health through the Food Quality Protection Act*, that points out flaws in the EPA testing process that have yielded a less than “complete and reliable database on developmental neurotoxicity of pesticides . . . upon which to base any final tolerance reassessment decisions as required by the FQPA.” Among other issues, they wrote, the EPA’s required pesticide testing does not include sufficient evaluation of behavior, learning, or memory in developing animals.

It is well known that acute high-level exposure to OP and carbamate pesticides can cause profound neurotoxicity, says Brenda Eskenazi, a professor at the University of California, Berkeley, and director of the NIEHS Center for Children’s Environmental Health Research there. But now there is some evidence that lower-level exposure to OPs could be linked to neonatal neurotoxicity, she says, and a number of studies are now being conducted on potential health consequences to older children.

Most people who are exposed to pesticides are exposed to more than one simultaneously, Eskenazi says, “so it’s really hard to say that a single agent is the ‘cause’ of an observed health problem in human epidemiologic studies.”

## Precaution and Progress

The EPA union leaders believe that such uncertainty is grounds for banning many of these pesticides under the precautionary principle, which advocates erring on the side of safety in the absence of full scientific certainty. In contrast, say some observers, the current FQPA reregistration process puts the onus on parties other than registrants to demonstrate that a pesticide is unsafe. “Until EPA can state with scientific confidence that these pesticides will not hurt the neurological development of our nation’s born and unborn children, there is no justification to continue the registration of the use of the remaining OP and carbamate pesticides,” the union leaders wrote.

The union letter also argued that the EPA failed in its risk analyses to consider the effects on farmworker families of agricultural pesticide application. According to the letter, the EPA’s analyses did not take into account that homes near agricultural fields may be exposed to pesticides that are not approved for home use.

But Lindsay responds that “we actually think that the way we do our risk assessments ensures that, in that scenario, kids and folks in the home will be safe.”

With the fate of just one pesticide yet unclear, the EPA sees its task as nearly done. “When we’ve done both the individual reassessment for aldicarb and then the cumulative for those five carbamates, we will have completed all of the FQPA tolerance reassessments,” Lindsay says. “It’s a real priority for us to get it completed.”

Hirzy, for one, doesn’t see the pesticide reassessments as a completed task, however. “[EPA officials] think they have dealt with our concerns that we raised in the letter, and we don’t think that they have,” he says. “I think we need to sit down with them . . . and reach some sort of agreement on how the agency will deal more forthrightly with specific concerns that have been raised on the record by EPA scientists.”

## Figures and Tables

**Figure:**